# PosMed: ranking genes and bioresources based on Semantic Web Association Study

**DOI:** 10.1093/nar/gkt474

**Published:** 2013-06-11

**Authors:** Yuko Makita, Norio Kobayashi, Yuko Yoshida, Koji Doi, Yoshiki Mochizuki, Koro Nishikata, Akihiro Matsushima, Satoshi Takahashi, Manabu Ishii, Terue Takatsuki, Rinki Bhatia, Zolzaya Khadbaatar, Hajime Watabe, Hiroshi Masuya, Tetsuro Toyoda

**Affiliations:** ^1^Bioinformatics and Systems Engineering Division (BASE), RIKEN, 1-7-22 Suehiro-cho, Tsurumi-ku, Yokohama, Kanagawa 230-0045, Japan, ^2^Integrated Database Unit, Advanced Center for Computing and Communication (ACCC), RIKEN, 2-1, Hirosawa, Wako, Saitama 351-0198, Japan and ^3^Technology and Development Unit for Knowledge Base of Mouse Phenotype, BioResource Center (BRC), RIKEN, 3-1-1 Koyadai, Tsukuba, Ibaraki 305-0074, Japan

## Abstract

Positional MEDLINE (PosMed; http://biolod.org/PosMed) is a powerful Semantic Web Association Study engine that ranks biomedical resources such as genes, metabolites, diseases and drugs, based on the statistical significance of associations between user-specified phenotypic keywords and resources connected directly or inferentially through a Semantic Web of biological databases such as MEDLINE, OMIM, pathways, co-expressions, molecular interactions and ontology terms. Since 2005, PosMed has long been used for *in silico* positional cloning studies to infer candidate disease-responsible genes existing within chromosomal intervals. PosMed is redesigned as a workbench to discover possible functional interpretations for numerous genetic variants found from exome sequencing of human disease samples. We also show that the association search engine enhances the value of mouse bioresources because most knockout mouse resources have no phenotypic annotation, but can be associated inferentially to phenotypes via genes and biomedical documents. For this purpose, we established text-mining rules to the biomedical documents by careful human curation work, and created a huge amount of correct linking between genes and documents. PosMed associates any phenotypic keyword to mouse resources with 20 public databases and four original data sets as of May 2013.

## INTRODUCTION

Mouse bioresources contribute to the study of human genes and diseases (1,2). To elucidate the function of all mouse genes, the International Knockout Mouse Consortium systematically generates mutant embryonic stem cells for every protein-coding gene (3), and the International Mouse Phenotype Consortium produces knockout mice and carries out high-throughput phenotyping of each line (4). Including other mouse resources, >24 000 mouse strains are registered in the International Mouse Strain Resource (IMSR) (5). To enhance the value of bioresources, we applied our original statistical search engine called the General and Rapid Association Study Engine (GRASE) and provided this as a web-oriented service called Positional MEDLINE (PosMed) (6–9). PosMed not only allows users to retrieve mouse bioresources directly with phenotypic keywords described in bioresource annotations, but also inferentially through corresponding documents for genes, diseases, drugs, ontologies, pathways, metabolites, molecular interactions and MEDLINE abstracts. With this inferential association search function, PosMed discovers wider resources than simple keyword search and accelerates the utilization of bioresources, especially those having fewer phenotypic annotations. In particular, knockout strains are not fully used when the targeted gene has an unknown function and no observed phenotype. PosMed connects these functionally unknown genes to known genes using molecular interactions, pathway information and/or co-citations and enables the suggestion of unobserved phenotypic bioresources as a search result.

**Figure 1.**
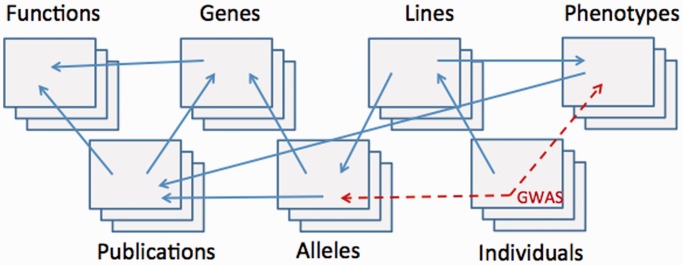
Concept of SWAS, which calculates the statistical significance of associations between any sets of resources connected through a web of semantic links (solid arrows), while GWAS associates only between alleles and phenotypes (dashed arrows).

PosMed is also applicable to the functional interpretation of genetic variants detected by exome sequencing studies. When users submit a list of genes and a phenotypic keyword, PosMed ranks the genes by statistical relevance between the keyword and each gene (6–9). These search functions are implemented using Semantic Web Association Study (SWAS) technology. The Semantic Web and linked data originally aim to provide a common framework that allows data to be shared and reused across application, enterprise and community boundaries (10,11). For most biological research purposes, however, association studies of linked data provide more analytical insights into biological systems than simple pattern-matching queries of the data (6). To take advantage of the Semantic Web of biological linked data, we propose to extend the methodology of association studies to the methodology called a ‘Semantic Web Association Study (SWAS)’ (Figure 1). A typical example of SWAS is the ‘Genome-wide Association Study (GWAS)’, which focuses only on the association between allelic variants and phenotypes in different individuals. In expanding the methodology of GWAS, SWAS explores more distant correlations among genes, functions, publications, alleles, lines, phenotypes and any subset specified by a user’s keywords. Because the conventional Semantic Web Resource Description Framework (RDF) (http://www.w3.org/RDF/) and query language SPARQL (http://www.w3.org/TR/rdf-sparql-query/) do not adequately support statistical evaluation of semantic links, we developed the GRASE for the implementation of PosMed (6,8).

### General usage of PosMed for bioresources

**Figure 2.**
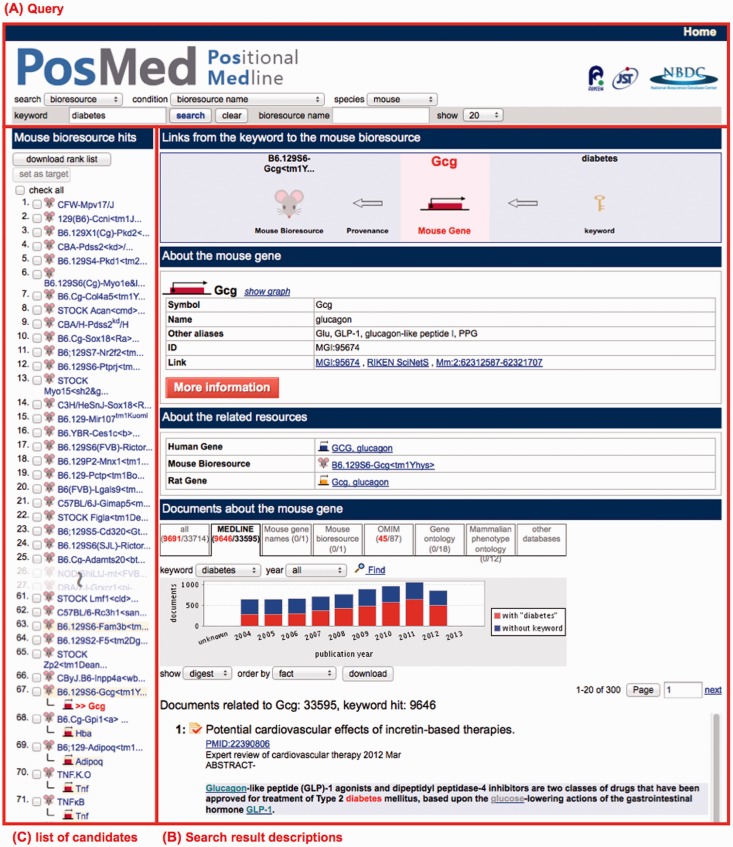
Example inferential search result followed by direct search results for retrieving a mouse bioresource associated with the keyword ‘diabetes’. PosMed shows the path connecting from a user’s keyword to the resource, a resource description and linked biological documents (B). To download all candidate mouse strains, click ‘check all’ at the top of ‘Hit resources’ and download them as a text file (C).

PosMed prioritizes genes, bioresources, diseases, metabolites or drugs depending on the statistical relevance between a user’s keyword and biological documents. The algorithm computing *P*-value is described in our previous publications (6,7). PosMed provides paths connecting the user’s keyword to the targeted resources. Figure 2 shows an example of two-step inferential or indirect search result associating a mouse resource with the keyword ‘diabetes’. Although the mouse strain ‘B6.129S6-Gcg<tm1Yhys>’ was not directly annotated with ‘diabetes’, PosMed suggested it via mouse gene ‘gcg, glucagon’, which has thousands of documents annotated with ‘diabetes’. PosMed provides up to three steps of inferential search function. For more examples such as specified genomic interval queries, please see the tutorial provided on our PosMed Web site.

### Advanced search with selection of biological documents and search paths

**Figure 3.**
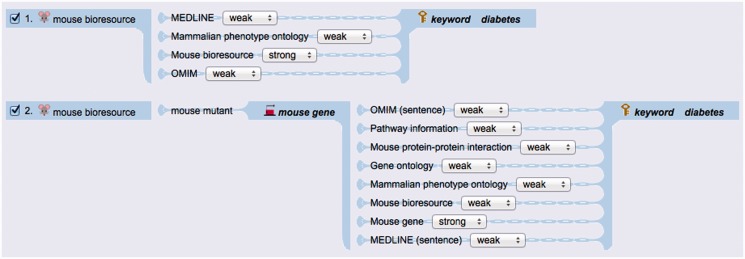
A partial example snapshot for ‘expert mode’. The upper path (1.) shows direct search with MEDLINE, mammalian phenotype ontology, mouse bioresources and OMIM documents. The lower path (2.) shows an example inferential path via gene. Users can select the scoring method of each document from ‘strong’, ‘weak’ or ‘none’ in the menu. The ‘strong’ scoring method uses a Boolean function and the *P*-value becomes 0 when the document has at least one keyword. The ‘weak’ method computes *P*-value using Fisher’s exact test. If a user selects ‘none’, the biological document is not used (6,7). In this mode users can confirm all PosMed search paths for biological documents.

PosMed provides several options for users to select the search paths, the documents used for the search, and the search scoring method from ‘expert mode’ of the advanced search setting page (Figure 3). With the expert mode menu, users can also select whether or not to use statistical significance association or Boolean association methods to associate biological items such as genes, chemicals and bioresources to a user’s query directly or indirectly through user-selected search paths. The statistical significance associates biological items based on the *P*-values using Fisher’s exact test of co-occurrence of the linked items in documents of OMIM, pathways, protein–protein interaction, gene ontology, phenotype ontology and other annotations, while the Boolean method associates the linked items co-occurring in the documents equally by ignoring the degree of significance (6).

### PosMed assists functional interpretation after exome sequencing

**Figure 4.**
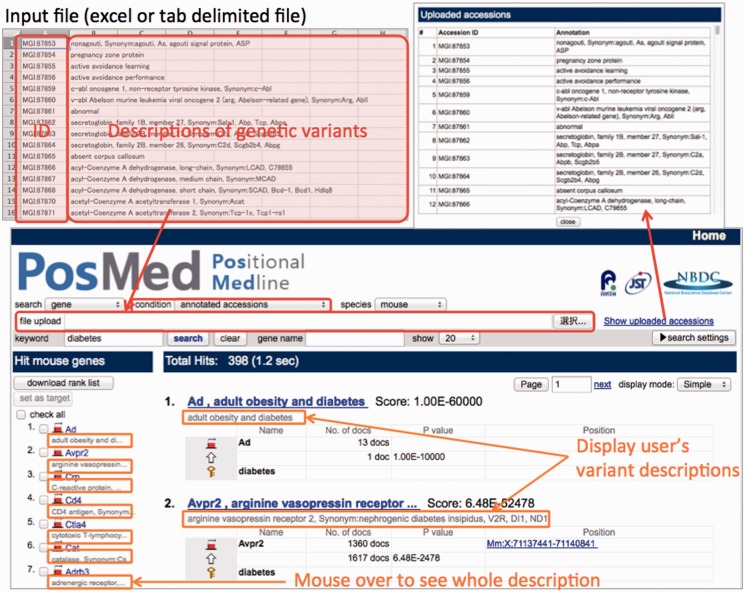
File upload function and display of users’ descriptions. Users can upload an excel file with gene IDs and descriptions by the user. PosMed ranks the genes listed within the files by statistical relevance between the user’s keyword and each gene, and displays the ranked genes together with the descriptions uploaded by the user.

Exome sequencing studies usually find several hundred to several thousand genetic variants by comparing samples and controls. To help prioritize the thousands of candidate genes for which PosMed calculates the ranking, PosMed accepts a list of gene IDs with the user’s descriptions of the gene variants. The descriptions in the uploaded file are displayed together with the ranked gene, allowing users to interpret the functionality of the gene variations (Figure 4). Detailed pages for each gene assist functional interpretation by showing biological documents such as MEDLINE, gene annotations, OMIM, bioresources, pathway information, molecular interactions, ontologies and links to related databases (Table 1).

**Table 1.** Updated biological documents for PosMed 2013

Document set	No. of documents	Data contents	References
Mouse bioresource	19 280	Mouse strain information registered at IMSR.	(5)
	5115	Mouse strain information from RIKEN BioResource center.	(12)
Human gene	37 287	Gene annotation of HGNC	(13)
Mouse gene	85 726	Gene annotation of MGI	(14)
Rat gene	36 634	Gene annotation of RGD	(15)
*Arabidopsis* gene	32 041	Gene annotation of TAIR	(16)
Rice gene	29 389	Gene annotation of RAP-DB	(17)
Disease	20 054	Online Mendelian Inheritance in Man	(18)
	2037	Manually collected our original data	(7)
	12 131	ICD-10, International Statistical Classification of Diseases and Related Health Problems	(19)
Metabolite	49 983	A comprehensive species-metabolite relationship database (KNApSAcK)	(20)
MEDLINE	9 378 134	MEDLINE titles, abstracts and MeSH terms	(21)
Pathway information	3809	Pathway information from REACTOME	(22)
Protein–protein interaction	73 645	Protein–Protein Interactions in Human and Mouse from rom IntAct and *Arabidopsis* from AtPID	(23,24)
Gene ontology	12 787	Gene ontology data	(25)
Human disease ontology	2282	Human disease ontology data	(26)
Mammalian phenotype ontology	7440	Mammalian phenotype ontology data	(27)

All data sources and links to the original DBs are described at http://omicspace.riken.jp/Data/.

### Extension of data coverage

Since previous publication, we updated 17 databases to include ∼10 million biological documents (Table 1; 7,9). To enhance the inferential search function for bioresources retrieval, we newly installed the following three biological documents: mammalian phenotype ontology (MP), human disease ontology (DO) and the International Classification of Diseases (ICD-10) and updated semantic links between each biomedical document. For example, we re-annotated mouse gene to MEDLINE by defining named entity recognition (NER) rules to retrieve correct publications (6–9). For human genes, we connected to publications via mouse homologs and newly defined NER rules for 2249 human non-homolog genes against mouse. Users can download these NER rules from our Web site.

In our previous publications, we used molecular interactions and co-expression to make links from fewer annotated biological resources to well annotated resources. These relationships are important to show more candidates. On the other hand, PosMed accuracy is strongly affected by low-quality data. Because Omics data are accumulated with various experimental methods, we selected high-quality data and removed low-trust data such as the classical yeast two-hybrid of protein–protein interaction (28).

For Semantic Web compliant data preparation, we used RIKENBASE or the RIKEN Scientists’ Networking System (SciNetS) (29), and public data are downloadable though Biophenome Linked Open Databases (BioLOD) (http://biolod.org). At least once a month we update PosMed data and its search index over the 10 million biological documents.

### Implementation

PosMed is implemented as a web application that users can access freely via their web browsers without log in. Although users can use a conventional web browser and a web browser plug-in is not needed, for Windows we recommend Microsoft Internet Explorer 9 or later, Firefox 18 or later and Google Chrome 24 or later. For Macintosh we recommend Safari 5 or later and Firefox 18 or later.

The web server is developed in Java and contains 11 Linux servers, including 10 distributed servers using GRASE engines (6) that perform direct search and inferential search in parallel, and one head server performing as both the Java Servlet user interface and the coordinator that evokes parallel search requests to the distributed servers and composes their results to rank the resultant data items. This architecture realizes scalability, so the search process can still be done in a few seconds even though our data sets are extended since our previous manuscript (7,9).

Although since the first launch of the PosMed service we have often been requested by users to implement a system to support batch queries, we do not support this yet because of machine resource limitations (PosMed consumes ∼1 to several seconds per query). Batch queries will be supported by securing additional machine resources in the future.

## DISCUSSION

Since 2005, PosMed has been widely used to prioritize candidate genes after Quantitative Trait Locus (QTL) analysis in mice and successfully identify responsible genes (30). This time, we added a file upload function (described in Figure 4) to modify the application of QTL analysis to exome sequencing studies. For data content, we added three new databases and ontologies to expand PosMed inferential search to bioresources. These data sets allow PosMed to discover bioresources with phenotypes, while most other databases only support genetic information. We expect our work to assist active use of bioresources.

Several eminent databases have released RDF files, but not so many scientists use Semantic Web technology actively. This may be partially because bioinformaticians like to calculate the statistical significance of associations of the RDF connections rather than a simple Boolean retrieval of the connections. To solve this problem, we propose our original methodology SWAS for statistical searching of the biological Semantic Web data. PosMed executes SWAS to rank significantly enriched groups of biological resource data items that can be associated with a user-specified query through the big data of Medline, omics data sets, other semantic data and so on. Our results confirm that such enrichment analysis using our SWAS methodology is effective (31) and provides many practical usage cases of enrichment studies including biological resource ranking problems. In the near future of big-data-driven science, the SWAS methodology needs to be added to the SPARQL end point services worldwide for any user to execute enrichment study over linked open data distributed around the world.

## FUNDING

National Bioscience Database Center (NBDC) of the Japan Science and Technology Agency (JST). Funding for open access charge: Grant from the NBDC of the JST. 

*Conflict of interest statement.* None declared.
